# Correction to: Spatiotemporal predictions of toxic urban plumes using deep learning

**DOI:** 10.1093/pnasnexus/pgag064

**Published:** 2026-04-24

This is a **correction** to:

Yinan Wang, M Giselle Fernández-Godino, Nipun Gunawardena, Donald D Lucas, Xiaowei Yue, Spatiotemporal predictions of toxic urban plumes using deep learning, *PNAS Nexus*, Volume 4, Issue 6, June 2025, pgaf198, https://doi.org/10.1093/pnasnexus/pgaf198

The following affiliation, Atmospheric, Earth and Environmental Division, Lawrence Livermore National Laboratory, Livermore, CA 94550, USA is incorrect and should be,

Atmospheric, Earth and Energy Division, Lawrence Livermore National Laboratory, Livermore, CA 94550, USA. This error has been corrected.

In addition, the **Acknowledgments** section statement has been corrected to read: “The authors thank A. Gowardhan from LLNL for providing data and information about the Aeolus model, LLNL's Data Science Summer Institute for partial support, and the anonymous reviewers for their valuable suggestion.” instead of: “The authors thank the anonymous reviewers for their valuable suggestions.”.

